# Global Trends and Disparities in Maternal Mortality: A Longitudinal Multi‐Country Analysis

**DOI:** 10.1002/hsr2.73478

**Published:** 2026-11-05

**Authors:** Awoke Fetahi Woudneh, Yenesew Fentahun Gebrie, Misganaw Mekonnen Nigussie, Kebadu Tadesse Cherie, Fetene Getnet Gebeyehu

**Affiliations:** ^1^ Department of Statistics Debremarkos University Debremarkos Ethiopia

**Keywords:** global health, HDI, health disparities, longitudinal analysis, maternal mortality, mixed‐effects model

## Abstract

**Background and Aims:**

Maternal mortality is one of the most significant global public health challenges and an important indicator of health‐system performance and socioeconomic development. Although substantial reductions have been achieved since 1990, progress has slowed in recent years, and substantial disparities persist across countries and regions.

**Methods:**

This longitudinal ecological study used country‐level panel data from the Maternal Mortality Dataset compiled by Banerjee (2021), covering 182 countries from 1990 to 2021. Maternal mortality ratio (MMR), expressed as deaths per 100,000 live births, was natural‐log transformed for statistical modeling. Human Development Index (HDI) group and continent were included as fixed‐effect predictors, while linear mixed‐effects models with country‐specific random intercepts and random slopes for centered year were used to account for between‐country heterogeneity and differences in temporal trajectories.

**Results:**

Maternal mortality declined over the study period (*β* = −0.029, 95% CI, −0.032 to −0.026; *p* < 0.001). Compared with the High HDI group, countries in the Low HDI group had higher log(MMR) (*β* = 1.803, 95% CI, 1.458–2.147; *p* < 0.001), as did countries in the Medium HDI group (*β* = 1.042, 95% CI, 0.767–1.317; *p* < 0.001), whereas the Very High HDI group had lower log(MMR) (*β* = −1.004, 95% CI, −1.268 to −0.739; *p* < 0.001). Compared with Africa, log(MMR) was lower in America (*β* = −0.488, 95% CI, −0.813 to −0.162; *p* = 0.004), Asia (*β* = −0.838, 95% CI, −1.134 to −0.542; *p* < 0.001), Europe (*β* = −1.698, 95% CI, −2.067 to −1.330; *p* < 0.001), and Oceania (*β* = −0.953, 95% CI, −1.404 to −0.502; *p* < 0.001). Adding random slopes improved model fit (*χ* ^2^ = 8041.6, df = 2; *p* < 0.001), indicating heterogeneity in country‐specific temporal trajectories. Marginal and conditional *R* ^2^ were 0.849 and 0.997, respectively.

**Conclusion:**

Maternal mortality declined substantially between 1990 and 2021, but disparities persisted across HDI groups, geographical regions, and countries. The findings highlight the importance of socioeconomic and geographical context when interpreting global maternal mortality patterns and support context‐specific approaches to further reduce maternal mortality.

## 1 Introduction

Maternal mortality is a key indicator of population health, reflecting the effectiveness of health systems and broader socioeconomic development. It is defined as the death of a woman during pregnancy, childbirth, or within 42 days of termination of pregnancy from causes related to or aggravated by the pregnancy or its management [1]. Despite sustained global efforts, maternal mortality continues to be a major public health challenge, particularly in low‐ and middle‐income countries.

Globally, substantial progress has been achieved over the past three decades. Maternal deaths declined from an estimated 532,000 in 1990 to approximately 287,000 in 2020, driven largely by global health initiatives such as the Millennium Development Goals (MDGs) [2, 3]. However, the pace of reduction has slowed in recent years, and the global maternal mortality ratio (MMR) remains far above the Sustainable Development Goal (SDG) target of fewer than 70 deaths per 100,000 live births by 2030 [3, 4]. This highlights the need for renewed efforts and evidence‐based strategies to accelerate progress.

Marked disparities persist across regions and countries. Sub‐Saharan Africa accounts for nearly 70% of global maternal deaths, followed by South Asia, while high‐income countries have achieved substantially lower MMR levels [3, 5]. These inequalities are largely attributed to differences in access to essential maternal health services, including skilled birth attendance, emergency obstetric care, and antenatal services [6, 7]. Importantly, heterogeneity in maternal mortality is not limited to differences between countries or broad geographical regions; substantial variation can also occur within individual countries. Recent ecological time‐series evidence from Türkiye, for example, demonstrated regional differences and temporal variability in maternal mortality between 2008 and 2023, highlighting the importance of considering both between‐country and within‐country heterogeneity when interpreting maternal mortality patterns [8]. Such disparities underscore the importance of examining overall trends as well as socioeconomic and geographical factors associated with differences in maternal mortality.

The factors associated with maternal mortality are complex and operate at multiple levels. Socioeconomic factors such as income, education, and poverty play a significant role in shaping maternal health outcomes [9, 10]. Higher levels of female education, for instance, are associated with increased utilization of maternal health services and improved health‐seeking behavior [11]. Demographic factors, including fertility rates, adolescent pregnancy, and short birth intervals, may further contribute to maternal health risks [12]. Recent demographic analyses suggest that changes in fertility, particularly among younger age groups, reflect not only broader demographic transitions but also changes in reproductive behavior [13, 14]. These demographic shifts are relevant to maternal mortality because changes in the level and age distribution of fertility can influence patterns of pregnancy and the population at risk. In addition, health system factors such as access to skilled health personnel, quality of care, and health financing are critical determinants of maternal survival [5, 6].

Weak health systems in many low‐resource settings contribute to delays in seeking, reaching, and receiving appropriate care, commonly conceptualized as the “three delays” model [15]. Broader structural factors, including inequality, governance, and health system quality, further influence maternal mortality outcomes [16]. These interrelated factors highlight the need for analytical approaches that account for both country‐level differences and temporal changes.

Although previous studies have provided valuable insights into maternal mortality trends, many have relied on cross‐sectional or short‐term analyses, limiting their ability to capture long‐term dynamics. Longitudinal multi‐country analyses provide a framework for examining both changes within countries over time and heterogeneity between countries [5, 17]. The period from 1990 to 2021 is particularly informative because it encompasses major global health and development transitions, including the MDG era and the subsequent SDG agenda. Examining this period therefore provides an opportunity to characterize long‐term changes in maternal mortality and differences associated with levels of human development and geographical context.

Therefore, this study aims to examine global trends and disparities in maternal mortality using longitudinal data from multiple countries between 1990 and 2021. Specifically, the study evaluates temporal changes in maternal mortality and examines how these patterns vary according to human development group and geographical region while accounting for country‐level heterogeneity through linear mixed‐effects modeling. By focusing on longitudinal patterns and population‐level associations, this study seeks to provide evidence that can contribute to understanding global inequalities in maternal mortality and accelerate progress toward reducing maternal mortality worldwide.

## 2 Materials and Methods

### 2.1 Study Design

The study design used was a longitudinal ecological study design, which aimed at examining the trends of maternal mortality and differences in maternal mortality over a period of 32 years (1990–2021) across multiple countries. The longitudinal design enabled the study of changes over time within countries, while the ecological design allowed comparison of cross‐country variation in MMRs across countries with different socioeconomic and geographical characteristics. This design offers a framework integrating the two dimensions (temporal and cross‐country) to examine population‐level associations between human development, geographical context, and maternal mortality. Furthermore, the multi‐country perspective allows for identification of regional patterns and global trends, which are critical for understanding disparities in maternal mortality and informing efforts to reduce maternal deaths. The study included 182 countries and 5824 country‐year observations over the 32‐year study period.

### 2.2 Data Sources

The analysis used the publicly available Maternal Mortality Dataset compiled by Banerjee (2021) and distributed through Kaggle. The dataset provides country‐year maternal mortality estimates compiled from international sources, including WHO, World Bank, and UNDP‐related information. Because the present study used the compiled country‐year dataset rather than independently reconstructing the estimates from the original international databases, the Kaggle dataset is treated as a secondary data source. The use of this source is acknowledged as a limitation, particularly with respect to version control, provenance, and potential differences in data processing from the original source databases.

### 2.3 Variables Included in the Current Investigation

The primary outcome of this study was the MMR, defined as the number of maternal deaths per 100,000 live births. Annual MMR data for each country from 1990 to 2021 were extracted from the dataset, enabling analysis of trends over time and differences between countries and regions. The main predictors included in the analysis were Human Development Index (HDI) group based on 2021 HDI information and continent, categorized as Africa, Asia, Europe, the Americas, and Oceania, to assess differences in maternal mortality according to development status and geographical region. The HDI classification used in this analysis was based on the 2021 HDI information and was treated as a time‐invariant country characteristic for the longitudinal analysis. Thus, the HDI group represents countries’ development status at the endpoint of the study period rather than a time‐varying measure of development throughout 1990–2021. Country was included as a random effect with both random intercepts and random slopes for centered year to account for unobserved differences in baseline maternal mortality and temporal trends across countries. Although other socioeconomic and health‐system indicators, such as GDP per capita, female education, fertility rate, skilled birth attendance, antenatal care coverage, urbanization, and health expenditure, may also be associated with maternal mortality, these were not included because they were not consistently available in the dataset. This combination of outcome and predictor variables provided a framework for examining global temporal trends and differences in maternal mortality according to human development and geographical context.

### 2.4 Data Structure

The data were structured as a longitudinal panel, where each observation was a country‐year, denoted by the ISO3 code and country name. This design enabled examination of temporal trends both between countries and regions and to compare countries. The panel format also allowed the use of linear mixed‐effects models, which allowed the study to explain the changes in maternal mortality over time as well as the country‐specific differences in mortality over the three decades.

### 2.5 Data Processing and Quality Control

The data were checked for duplicate records, missing values, inconsistent country identifiers, and invalid or non‐positive MMR values. MMR values were converted to numeric format, and annual observations were reshaped into a country‐year longitudinal format. Because the statistical model used the natural logarithm of MMR, observations with missing or non‐positive MMR values were excluded from the modeling dataset. The calendar year was centered at 1990 by calculating ${\mathrm{Year}}_{t}^{*}=$ ${\mathrm{Yeart}}_{t}$ − 1990. The resulting dataset was checked for consistency of country‐year observations before statistical modeling.

### 2.6 Statistical Analysis

All statistical analyses were conducted using R software (version 4.5.1) [18]. Maternal mortality trends were explored graphically, using line plots to illustrate temporal changes in the MMR from 1990 to 2021 at both the population and country levels.

To examine longitudinal patterns and population‐level differences in maternal mortality while accounting for both temporal and country‐level variation, linear mixed‐effects models (LMMs) were fitted using the lme4 package in R [19]. The model used the natural logarithm of the MMR (log(MMR)) as the outcome variable. A natural log transformation was applied because the distribution of MMR was strongly right‐skewed, and the transformation reduced the influence of extreme values and provided a more suitable scale for the linear mixed‐effects analysis. The model included log(MMR) as the outcome variable, HDI group, centered year, and continent as fixed‐effect predictors, and country as a random effect with both random intercepts and random slopes for centered year to capture unobserved heterogeneity in baseline maternal mortality and temporal trends across countries.

### 2.7 Model Diagnostics

The assumptions of the linear mixed‐effects model were assessed using graphical diagnostics. In particular, the distribution of the model residuals was examined using a histogram with an overlaid normal density curve. The residuals were approximately symmetric and centered on zero, with a broadly bell‐shaped distribution and minor deviations in the tails (Figure S1). Based on this assessment, the residual distribution was considered reasonably consistent with the normality assumption of the linear mixed‐effects model.

The model can be expressed mathematically as:

$$\log\left({\mathrm{MMR}}_{\mathrm{it}}\right)=\beta_{0}+\beta_{1}{\mathrm{Year}}_{t}^{*}+\beta_{2}{\mathrm{HDI}}_{i}+\beta_{3}{\mathrm{Continent}}_{i}+u_{0i}+u_{1i}{\mathrm{Year}}_{t}^{*}+\epsilon_{\mathrm{it}}$$

where $\log\left({\mathrm{MMR}}_{\mathrm{it}}\right)$ is the natural logarithm of the MMR ratio for country i at year t; ${\mathrm{Year}}_{t}^{*}=\mathrm{Year}t_{t}$ − 1990 means that the calendar year is centered at 1990; $\beta_{0}\;$is the overall intercept; $\beta_{1}\;$is the fixed‐effect coefficient for centered year; $\beta_{2}\;$ and $\beta_{3}\;$are fixed‐effect coefficients for HDI and continent; $u_{0i}\sim N(0,\sigma_{u0}^{2})$ is the random intercept for country i; $u_{1i}\sim N(0,\sigma_{u1}^{2})$ is the country‐specific random slope for centered year, allowing the temporal trend to vary across countries; and $\epsilon_{\mathrm{it}}\sim N(0,\sigma^{2})$ is the residual error

Model selection was conducted by comparing a random‐intercept model with a random‐intercept/random‐slope model. The models were compared using likelihood ratio testing, with Akaike information criterion (AIC) and Bayesian information criterion (BIC) also considered. The random‐intercept/random‐slope model was retained as the final model because it provided a substantially better fit to the data. Following model selection, the final model was refitted using restricted maximum likelihood (REML). Fixed‐effect estimates were reported as regression coefficients (*β*) with standard errors (SEs) and 95% confidence intervals (CIs). Confidence intervals were calculated using Wald's method.

Likelihood ratio testing was used to compare the nested random‐intercept and random‐intercept/random‐slope models. The random‐effects structure was therefore selected based on model fit rather than by comparing a full model containing all possible interactions. All hypothesis tests were two‐sided, with an a priori significance level of 0.05. *p* values were reported as *p* < 0.001 when below 0.001, to three decimal places for values between 0.001 and 0.01, and to two decimal places for values ≥ 0.01. Statistical analyses were conducted using R version 4.5.1 with the lme4 and lmerTest packages [19]. Statistical reporting was guided by established recommendations for reporting clinical research statistics [20].

## 3 Results

### 3.1 Population and Country‐Level MMR Trends

Figure 1 demonstrates substantial heterogeneity in maternal mortality trajectories across the 20 randomly selected countries from 1990 to 2021. Although the overall pattern was one of declining MMR, countries differed markedly in their baseline levels and rates of decline. Countries with initially higher MMR generally showed substantial reductions over the study period, while several countries with lower initial MMR also experienced continued declines, resulting in persistent differences between countries by 2021. The black line, representing the mean MMR across the sampled countries, shows a progressive decline over time. These heterogeneous trajectories indicate that a single population‐average trend does not fully describe the country‐level patterns, providing a rationale for modeling both country‐specific baseline differences and country‐specific temporal slopes.

**Figure 1:**
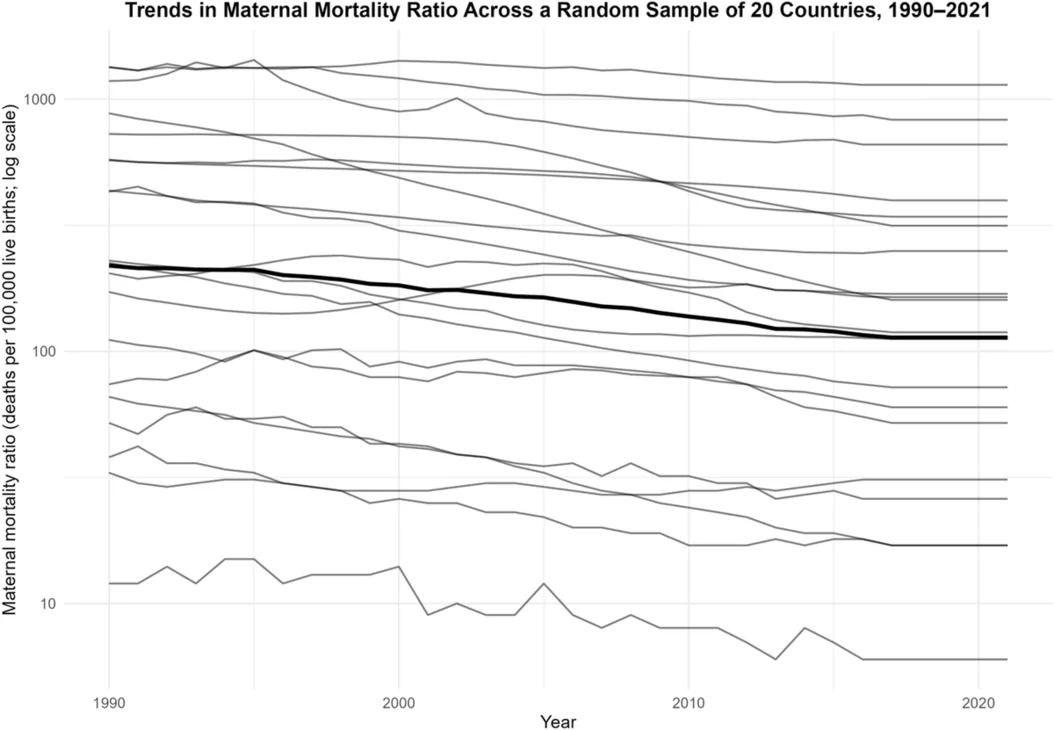
Maternal mortality trends across a random sample of 20 countries, 1990–2021.

Figure 2 illustrates the fixed‐effect predicted MMR from 1990 to 2021, presented on a logarithmic scale. The predicted MMR shows a clear and consistent downward trend over the study period, indicating an overall decline in maternal mortality after accounting for the fixed‐effect predictors included in the model. The approximately linear decline on the logarithmic scale suggests a relatively consistent proportional reduction in predicted MMR over time. Overall, the figure demonstrates substantial progress in reducing maternal mortality across the study period, while the model‐based trajectory represents the temporal pattern estimated from the fixed effects rather than country‐specific trajectories.

**Figure 2:**
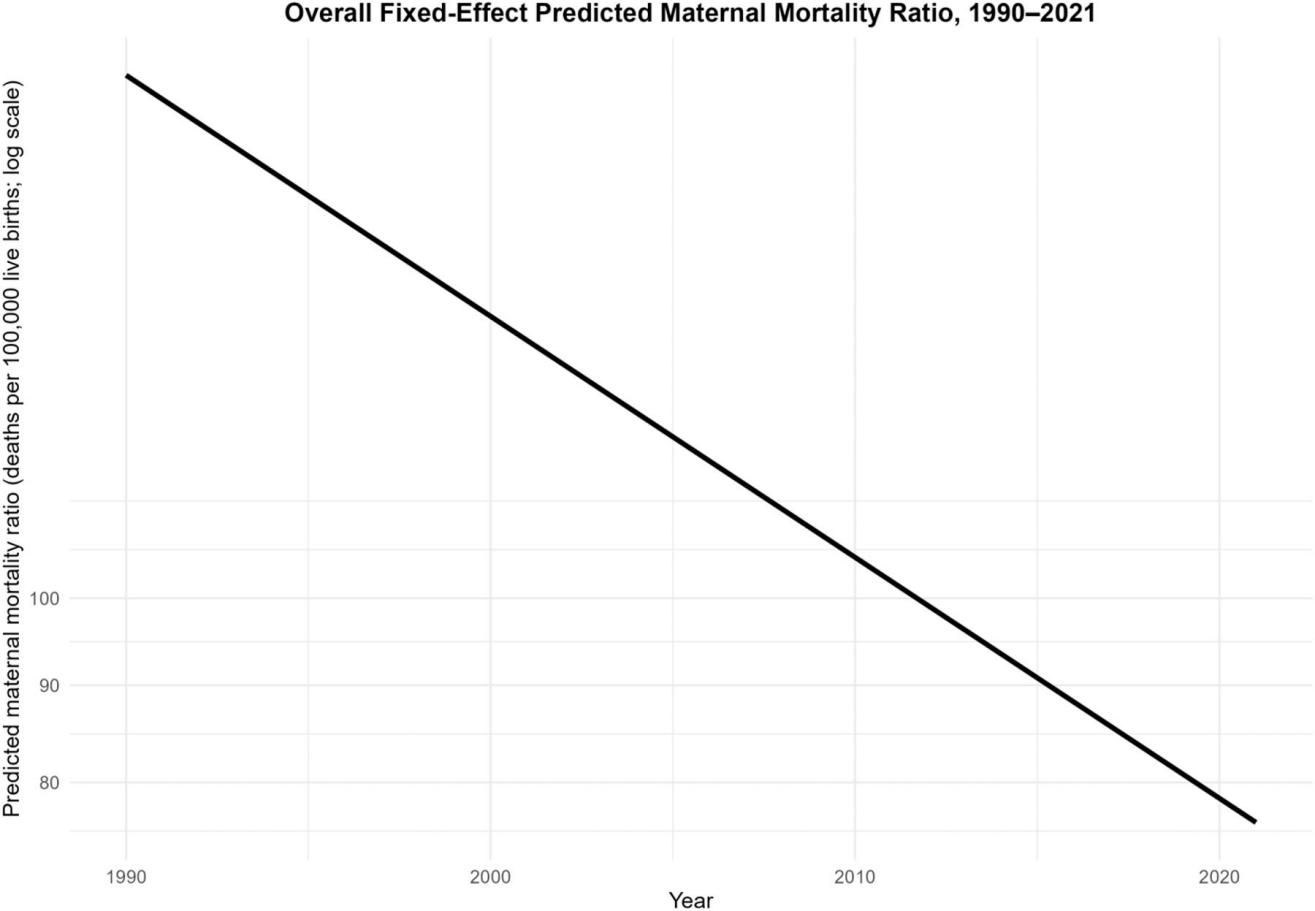
Overall fixed‐effect predicted maternal mortality ratio, 1990–2021.

Figure 3 presents the fixed‐effect predicted MMR from 1990 to 2021 across Human Development Index (HDI) groups, with the outcome displayed on a logarithmic scale. A consistent decline in predicted MMR is observed across all HDI groups, indicating an overall downward temporal trend in maternal mortality throughout the study period. The Low HDI group has the highest predicted MMR, followed by the Medium and High HDI groups, whereas the Very High HDI group has the lowest predicted MMR throughout the study period. This persistent separation between HDI groups indicates substantial differences in predicted maternal mortality according to development status. The approximately parallel downward trajectories suggest that the temporal decline is broadly similar across HDI groups, while the differences between groups remain evident over time. Because the predictions are presented on a logarithmic scale, the figure emphasizes relative rather than absolute changes in MMR across HDI groups.

**Figure 3:**
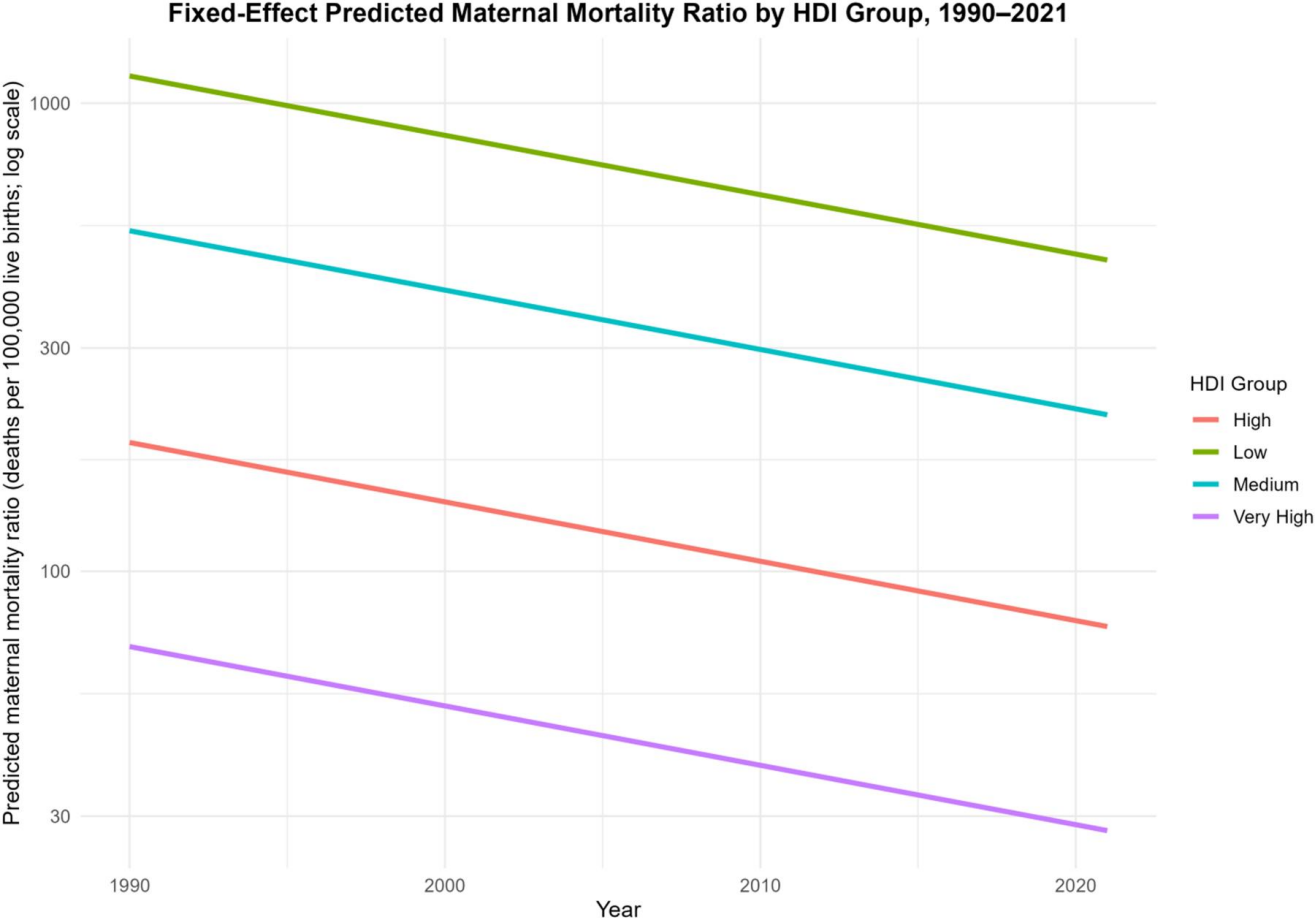
Fixed‐effect predicted maternal mortality ratio by HDI Group, 1990–2021.

### 3.2 Linear Mixed Model Estimates

As shown in Table 1, the random‐effects model illustrates considerable heterogeneity in maternal mortality rates among countries. As seen from both models, the variance in the random intercept is a clear sign of the fact that there are significant differences in the baseline levels of MMR among countries. However, Model 2 demonstrates even greater heterogeneity due to the presence of the variance of the random slope for time and the correlation of intercepts and slopes in Model 2. Namely, it can be assumed that countries with initially high MMR have a greater rate of reduction in mortality.

**Table 1:** Random effects and variance components.

	Model 1	Model 2
Random effect	Variance (SD)	Variance (SD)
Country (intercept)	0.390 (0.624)	0.518 (0.720)
Country (year slope)	—	0.0004 (0.020)
Residual	0.045 (0.212)	0.009 (0.096)
Intercept–slope correlation	—	−0.50

Table 2 presents the fixed‐effect estimates from the two linear mixed‐effects models. In both models, centered year was negatively associated with log(MMR), indicating a decline in maternal mortality over time (Model 1: *β* = −0.029, 95% CI, −0.030 to −0.029; *p* < 0.001; Model 2: *β* = −0.029, 95% CI, −0.032 to −0.026; *p* < 0.001). Compared with countries in the High HDI group, countries in the Low HDI group had higher log(MMR) (Model 2: *β* = 1.803, 95% CI, 1.458–2.147; *p* < 0.001), as did countries in the Medium HDI group (*β* = 1.042, 95% CI, 0.767–1.317; *p* < 0.001). In contrast, countries in the Very High HDI group had lower log(MMR) than the High HDI reference group (*β* = −1.004, 95% CI, −1.268 to −0.739; *p* < 0.001). Compared with Africa, all other continental groups had lower log(MMR), with the largest difference observed for Europe (*β* = −1.698, 95% CI, −2.067 to −1.330; *p* < 0.001), followed by Oceania (*β* = −0.953, 95% CI, −1.404 to −0.502; *p* < 0.001), Asia (*β* = −0.838, 95% CI, −1.134 to −0.542; *p* < 0.001), and America (*β* = −0.488, 95% CI, −0.813 to −0.162; *p* = 0.004). The fixed‐effect estimates were similar between the two models, indicating that inclusion of the country‐specific random slope did not materially alter the estimated fixed‐effect associations.

**Table 2:** Fixed‐effect estimates from the linear mixed‐effects models.

	Model 1			Model 2		
Variable	*β* (SE)	95% CI	*p*	*β* (SE)	95% CI	*p*
Intercept	5.223 (0.150)	4.929, 5.517	< 0.001	5.239 (0.152)	4.941, 5.537	< 0.001
Year (centered)	−0.029 (0.003)	−0.030, −0.029	< 0.001	−0.029 (0.0015)	−0.032, −0.026	< 0.001
HDI group						
Low	1.813 (0.176)	1.467,2.158	< 0.001	1.803 (0.176)	1.458, 2.147	< 0.001
Medium	1.055 (0.141)	0.780, 1.331	< 0.001	1.042 (0.140)	0.767, 1.317	< 0.001
Very high	−1.024 (0.136)	−1.290, −0.759	< 0.001	−1.004 (0.135)	−1.268, −0.739	< 0.001
Continent						
America	−0.492 (0.167)	−0.818, −0.166	0.003	−0.488 (0.166)	−0.813, −0.162	0.004
Asia	−0.804 (0.151)	−1.100, −0.507	< 0.001	−0.838 (0.151)	−1.134, −0.542	< 0.001
Europe	−1.655 (0.189)	−2.025,−1.286	< 0.001	−1.698 (0.188)	−2.067, −1.330	< 0.001
Oceania	−0.939 (0.231)	−1.392, −0.487	< 0.001	−0.953 (0.230)	−1.404, −0.502	< 0.001

*Note:* The outcome variable was log‐transformed MMR. High HDI was the reference category for HDI group, and Africa was the reference category for continent. All confidence intervals are 95% Wald confidence intervals. *p* values are two‐sided.

Abbreviations: *β*, fixed‐effect coefficient; CI, confidence interval; SE, standard error.

According to Table 3, the correlation matrix of the fixed‐effect estimates showed that none of the pairwise correlations exceeded 0.8, a commonly used threshold for indicating potentially problematic multicollinearity. The largest correlations were observed between the model intercept and the low HDI group (−0.764) and medium HDI group (−0.609), which are expected in part because categorical predictors are represented using dummy variables. The correlations among the other fixed‐effect predictors were generally moderate or low, while the correlation between centered year and the other fixed‐effect predictors was negligible (0.000). Overall, the correlation matrix did not indicate substantial multicollinearity among the fixed‐effect estimates.

**Table 3:** Correlation matrix of fixed‐effect estimates.

	Intr	Yr_cnt	HDI_GL	HDI_GM	HDI_GH	ContinentAm	ContinentAs	ContinentEu	ContinentOc
Year_centered	−0.176								
HDI_GroupLow	−0.764	0.000							
HDI_GroupMedium	−0.609	0.000	0.503						
HDI_GroupVery High	−0.289	0.000	0.263	0.378					
ContinentAmerica	−0.690	0.000	0.519	0.279	−0.033				
ContinentAsia	−0.669	0.000	0.481	0.197	−0.149	0.596			
ContinentEurope	−0.607	0.000	0.458	0.263	−0.374	0.578	0.632		
ContinentOceania	−0.420	0.000	0.321	0.054	−0.041	0.374	0.400	0.365	

*Note:* Africa is the reference continent.

Abbreviations : ContinentAm, America; ContinentAs, Asia; ContinentEu, Europe; ContinentOc, Oceania; DI_GH, high HDI group (reference); HDI_GL, low HDI group; HDI_GM, medium HDI group; HDI_GV, very high HDI group; Intr, model intercept; Yr_cnt, centered year

Table 4. The mixed‐effects model had a marginal *R* ^2^ of 0.849 and a conditional *R* ^2^ of 0.997. The large difference between marginal and conditional *R* ^2^ indicates that country‐level random effects account for a substantial proportion of the observed variability. The conditional *R* ^2^ should therefore be interpreted as a measure of variance explained by the combined fixed‐ and random‐effects structure rather than as evidence that the model has 99.7% predictive accuracy. The adjusted ICC was 0.979, indicating substantial clustering after accounting for the fixed effects, whereas the unadjusted ICC was 0.148. These findings indicate that country‐level heterogeneity contributes substantially to the observed variation in maternal mortality.

**Table 4:** Model fit statistics for the mixed‐effects model.

Statistic	Value
Adjusted ICC	0.979
Unadjusted ICC	0.148
Marginal *R* ^2^	0.849
Conditional *R* ^2^	0.997

Abbreviations: ICC, Intraclass correlation coefficient accounting for fixed effects.

Residual diagnostics indicated that the normality assumption was reasonably satisfied. The residuals were approximately symmetric and bell‐shaped and centered on zero, with minor deviations in the tails (Figure S1).

Table 5 shows that the random‐intercept/random‐slope model (Model 2) provided a substantially better fit than the random‐intercept model (Model 1), as indicated by the likelihood ratio test (χ^2^ = 8041.6, df = 2, *p* < 0.001) and the lower AIC and BIC values for Model 2. The results indicate that the temporal trajectory of maternal mortality varied across countries, supporting the inclusion of country‐specific random slopes for centered year in the final model.

**Table 5:** Comparison of linear mixed‐effects models predicting log(MMR).

Model	Random‐effects structure	npar	AIC	BIC	logLik	− 2 × log(L)	χ^2^	df	*p*
Model 1	Random intercept (1|country)	11	−525.3	−452.0	273.7	−547.3	_	_	_
Model 2	Random intercept + random slope (Year_centered|country)	13	−8562.9	−8476.2	4294.5	−8588.9	8041.6	2	< 0.001

*Note:* Lower AIC and BIC values indicate better relative model fit. Model 2 includes a country‐specific random intercept and random slope for centered year.

Abbreviations: AIC, Akaike information criterion; BIC, Bayesian information criterion; df, degrees of freedom; logLik, log‐likelihood; Npar, number of estimated parameters; χ^2^, likelihood ratio test statistic comparing Model 2 with Model 1.

Table 6, Comparison of model fit statistics, demonstrates that Model 2 provides a substantially better fit to the data than Model 1, as indicated by the markedly lower REML criterion (−8558.4 vs. −513.7). Both models are based on the same number of observations and countries; therefore, the improvement in fit can be attributed to the inclusion of the random slope for time. This finding supports the importance of allowing temporal trends to vary across countries when modeling maternal mortality, as it captures additional complexity in the data structure.

**Table 6:** Model fit statistics.

Statistic	Model 1	Model 2
Observations	5824	5824
Countries	182	182
REML criterion	−513.7	−8558.4

## 4 Discussion

This study examined longitudinal trends and disparities in maternal mortality between 1990 and 2021, demonstrating substantial progress alongside persistent inequalities. In the final mixed‐effects model, centered year was negatively associated with log(MMR) (*β* = −0.029, 95% CI, −0.032 to −0.026; *p* < 0.001), indicating an overall decline in maternal mortality over the study period. These results are consistent with global estimates indicating that maternal mortality has declined substantially over the last 30 years [3, 21]. Nonetheless, the rate of decline has been decreasing over the last few years in line with evidence that progress has stalled in many low‐resource contexts, making it difficult to meet targets of SDG targets [3, 4].

An important finding of this study was the association between socioeconomic development, as represented by the Human Development Index (HDI) group, and maternal mortality. Compared with the High HDI reference group, countries in the Low HDI group had higher log(MMR) (*β* = 1.803, 95% CI, 1.458–2.147; *p* < 0.001), as did countries in the Medium HDI group (*β* = 1.042, 95% CI, 0.767–1.317; *p* < 0.001). In contrast, countries in the Very High HDI group had lower log(MMR) than the High HDI reference group (β = −1.004, 95% CI, −1.268 to −0.739; *p* < 0.001). These findings are consistent with previous research linking socioeconomic conditions, education, income, living standards, and access to maternal health services with maternal health outcomes. Greater access to family planning and contraceptive services may also contribute to reductions in maternal mortality by preventing unintended and potentially high‐risk pregnancies [22]. However, because HDI was based on 2021 status and treated as a time‐invariant characteristic in the present analysis, these findings represent differences in maternal mortality according to endpoint development classification rather than evidence that changes in HDI caused changes in maternal mortality. These results underscore the importance of comprehensive strategies that focus on social determinants and health care access.

Inequality in the distribution of maternal mortality across geographical regions was also observed. Compared with Africa, the reference continent, all other continental groups had lower estimated log(MMR): America (*β* = −0.488, 95% CI, −0.813 to −0.162; *p* = 0.004), Asia (*β* = −0.838, 95% CI, −1.134 to −0.542; *p* < 0.001), Europe (*β* = −1.698, 95% CI, −2.067 to −1.330; *p* < 0.001), and Oceania (*β* = −0.953, 95% CI, −1.404 to −0.502; *p* < 0.001). This pattern is consistent with international reports showing that sub‐Saharan Africa accounts for a disproportionate share of global maternal deaths [3]. Differences in socioeconomic conditions, health‐system capacity, access to skilled birth attendance, emergency obstetric care, and health infrastructure may contribute to these geographical disparities. However, these factors were not directly measured in the present analysis and should therefore be considered possible explanations rather than direct findings of this study. Evidence indicates that skilled birth attendance and timely access to emergency obstetric care are important components of MMR [6, 23]. Moreover, local studies in sub‐Saharan Africa have revealed that the constraints of the health systems, such as the shortage of the workforce and insufficient funds, have been critical issues in enhancing maternal health outcomes [24].

The mixed‐effects analysis also demonstrated substantial heterogeneity between countries in both baseline maternal mortality and temporal trajectories. The inclusion of country‐specific random slopes indicated that the rate of change in log(MMR) varied across countries. The estimated correlation between the country‐specific random intercepts and slopes was −0.50, suggesting that countries with higher estimated baseline log(MMR) tended to have more negative estimated temporal slopes. This pattern is consistent with some degree of convergence in maternal mortality trajectories, although the remaining between‐country variability indicates that convergence was incomplete. These findings are consistent with international evidence showing that maternal mortality transitions have not been uniform across countries [3, 21]. The mechanisms underlying differences in country‐specific trajectories cannot be established from the present analysis, but may relate to differences in socioeconomic conditions, health‐system capacity, demographic characteristics, and policy implementation.

The variance and model *R* ^2^ estimates further demonstrated the importance of country‐level heterogeneity. The marginal *R* ^2^ was 0.849, whereas the conditional *R* ^2^ was 0.997, indicating that the fixed effects explained a substantial proportion of the variance in log(MMR), while the inclusion of country‐level random effects accounted for additional variation. The adjusted ICC was 0.979, indicating substantial clustering after accounting for the fixed effects. The large difference between marginal and conditional *R* ^2^ emphasizes the contribution of country‐level heterogeneity to the observed variation in maternal mortality. Importantly, the conditional *R* ^2^ represents variance explained by the combined fixed‐ and random‐effects structure and should not be interpreted as 99.7% predictive accuracy. Although differences in health‐system capacity and quality of care may contribute to country‐level heterogeneity, these factors were not directly measured in the present model and therefore cannot be identified as independent explanatory factors from these results. Previous research has nevertheless emphasized the importance of health‐system quality [16], access to maternal health services [23], and broader health‐system and socioeconomic conditions [24] for maternal health outcomes.

Although this study has strengths, including the use of a large longitudinal dataset and linear mixed‐effects modeling, several limitations should be acknowledged. First, the analysis relied on a secondary Kaggle‐compiled dataset rather than independently reconstructing estimates from the original international databases, which may introduce limitations related to data provenance, versioning, and harmonization. Second, the analysis included a relatively limited set of explanatory variables. Although HDI and continent capture important dimensions of socioeconomic and geographical heterogeneity, other potentially important determinants, including fertility, female education, health expenditure, skilled birth attendance, antenatal care, and economic conditions, were not included because of data availability and consistency across countries and years. Consequently, the estimated associations should not be interpreted as an exhaustive assessment of the factors associated with maternal mortality. Third, HDI was treated as a time‐invariant characteristic based on 2021 status. Consequently, countries were classified according to their endpoint development status throughout the study period. This approach does not capture changes in human development between 1990 and 2021 and may introduce endpoint classification bias when interpreting associations with earlier maternal mortality. Future studies should incorporate time‐varying HDI measures to better capture changes in development status over time. Fourth, the ecological design limits individual‐level interpretation and causal inference. The observed associations should therefore be interpreted as population‐level relationships rather than causal effects. Furthermore, differences in data quality and reporting across countries may contribute to measurement variability.

In conclusion, this study demonstrates that maternal mortality declined substantially worldwide between 1990 and 2021, although the magnitude and pace of progress varied across countries, HDI groups, and geographical regions. Differences in socioeconomic development and geographical context were associated with substantial variation in maternal mortality, while considerable country‐level heterogeneity remained in both baseline levels and temporal trajectories. Further reductions in maternal mortality will require sustained attention to health‐system capacity, access to quality maternal health services, and broader socioeconomic conditions. Future research should incorporate time‐varying HDI and more detailed demographic, health‐system, and maternal health service indicators to better understand the pathways underlying differences and changes in maternal mortality and to inform efforts to accelerate progress toward global maternal health goals.

## 5 Conclusion and Recommendation

### 5.1 Conclusion

In conclusion, this study demonstrates that maternal mortality declined substantially worldwide between 1990 and 2021, although the magnitude and pace of progress varied across countries, HDI groups, and geographical regions. Countries in lower HDI groups had higher estimated maternal mortality than those in the High HDI reference group, whereas countries in the Very High HDI group had lower estimated maternal mortality. Substantial geographical disparities were also observed, with African countries having higher estimated maternal mortality than the other continental groups.

The mixed‐effects analysis demonstrated substantial between‐country heterogeneity in both baseline maternal mortality and temporal trajectories. The inclusion of country‐specific random slopes indicated that rates of change in maternal mortality varied across countries, while the negative correlation between country‐specific random intercepts and slopes suggested some degree of convergence, with countries having higher estimated baseline log(MMR) tending to have more negative estimated temporal slopes. However, substantial between‐country variability remained throughout the study period.

Overall, these findings indicate that although maternal mortality declined substantially globally, disparities across development levels, geographical regions, and countries persisted. The findings highlight the importance of considering socioeconomic and geographical context when interpreting global maternal mortality patterns. Continued efforts to strengthen health systems and improve access to quality maternal health services, together with attention to broader socioeconomic conditions, may contribute to further reductions in maternal mortality. Future research should incorporate time‐varying HDI and more detailed demographic, socioeconomic, health‐system, and maternal health service indicators to better characterize the factors associated with differences in maternal mortality and changes over time.

### 5.2 Recommendation

Based on these findings, efforts to reduce maternal mortality should prioritize countries with lower HDI, where estimated maternal mortality was higher. Strategies should focus on improving access to essential maternal health services and addressing relevant socioeconomic conditions.

The variation in country‐specific trends suggests that interventions should be adapted to national contexts. Countries with slower declines may benefit from targeted assessment and context‐specific interventions.

The higher maternal mortality observed in Africa highlights the importance of strengthening maternal health systems and access to quality care in the region.

Broader socioeconomic development should also be considered alongside health interventions when addressing maternal mortality disparities.

Finally, future research should incorporate additional demographic, socioeconomic, health‐system, and maternal health service indicators to better explain variation in maternal mortality across countries.

## Author Contributions

**Awoke Fetahi Woudneh:** conceptualization, methodology, software, data curation, formal analysis, visualization, project administration, writing – original draft, writing – review and editing. **Yenesew Fentahun Gebrie:** conceptualization, methodology, investigation, validation, writing – review and editing. **Misganaw Mekonnen Nigussie:** formal analysis, data curation, validation, writing – review and editing. **Kebadu Tadesse Cherie:** investigation, writing – review and editing, data curation, validation. **Fetene Getnet Gebeyehu:** investigation, writing – review and editing, data curation.

## Funding

The authors have nothing to report.

## Ethics Statement

The authors have nothing to report.

## Consent

The authors have nothing to report.

## Conflicts of Interest

The authors declare no conflicts of interest.

## Transparency Statement

The corresponding author (Awoke Fetahi Woudneh) affirms that this manuscript is an honest, accurate, and transparent account of the study being reported; that no important aspects of the study have been omitted; and that any discrepancies from the study as planned have been explained.

## Supporting information


Supporting File

## Data Availability

The dataset used in this study is publicly available from Kaggle: Maternal Mortality Dataset: A Time‐Stratified Exploration by Sourav Banerjee (2021), accessible at https://www.kaggle.com/datasets/souravbanerjee/maternal-mortality-dataset

## References

[1] World Health Organization , Maternal mortality: Levels and trends 2000 to 2017. Geneva, 2019.

[2] Institute for Health Metrics and Evaluation . Global Burden of Disease Study 2019 Results. Seattle, WA, 2020.

[3] World Health Organization, United Nations Children's Fund, United Nations Population Fund, World Bank Group, United Nations Department of Economic and Social Affairs PD ., Trends in Maternal Mortality 2000 to 2020: Estimates by WHO, UNICEF, UNFPA (Geneva: World Bank Group and UNDESA/Population Division, 2023. Report No.: 978−92‐4‐006875‐9).

[4] Transforming Our World.” The 2030 Agenda for Sustainable Development (United Nations, 2015).

[5] N. J. Kassebaum , R. M. Barber , Z. A. Bhutta , et al., “Global, Regional, and National Levels of Maternal Mortality, 1990–2015: A Systematic Analysis for the Global Burden of Disease Study 2015,” Lancet 388, no. 10053 (2016): 1775–1812, 10.1016/S0140-6736(16)31470-2. PMC5224694 27733286

[6] O. M. Campbell and W. J. Graham , “Strategies for Reducing Maternal Mortality: Getting on With What Works,” Lancet 368, no. 9543 (2006): 1284–1299, 10.1016/S0140-6736(06)69381-1. 17027735

[7] L. Say , D. Chou , A. Gemmill , et al., “Global Causes of Maternal Death: A WHO Systematic Analysis,” Lancet Global Health 2, no. 6 (2014): e323–e333, 10.1016/S2214-109X(14)70227-X. 25103301

[8] B. I. Aylı , “Regional Convergence and Temporal Volatility in Maternal Mortality: An Ecological Time‐Series Analysis From Türkiye, 2008–2023,” Global Social Welfare, ahead of print, January 13, 2026, 10.1007/s40609-026-00438-5.

[9] W. Bank , World Development Indicators (World Bank, 2025).

[10] V. Filippi , C. Ronsmans , O. M. Campbell , et al., “Maternal Health in Poor Countries: The Broader Context and a Call for Action,” Lancet 368, no. 9546 (2006): 1535–1541, 10.1016/S0140-6736(06)69384-7. 17071287

[11] E. Gakidou , K. Cowling , R. Lozano , and C. J. Murray , “Increased Educational Attainment and Its Effect on Child Mortality in 175 Countries Between 1970 and 2009: A Systematic Analysis,” Lancet 376, no. 9745 (2010): 959–974, 10.1016/S0140-6736(10)61257-3. 20851260

[12] S. Ahmed , Q. Li , L. Liu , and A. O. Tsui , “Maternal Deaths Averted by Contraceptive Use: An Analysis of 172 Countries,” Lancet 380, no. 9837 (2012): 111–125, 10.1016/S0140-6736(12)60478-4. 22784531

[13] B. I. Aylı , “Beyond the Demographic Transition: Regional Convergence and Structural Shifts in Adolescent Fertility Trends in Türkiye, 2009–2024,” China Population and Development Studies 10, no. 1 (2026): 21–39, 10.1007/s42379-026-00212-2.

[14] B. I. Aylı and N. Paksoy , “Declining Fertility in Türkiye (2000−2023): A Kitagawa Decomposition Analysis Interpreted Through a Gender Lens,” Women's Studies International Forum 120 (2027): 103441, 10.1016/j.wsif.2026.103441.

[15] S. Thaddeus and D. Maine , “Too Far to Walk: Maternal Mortality in Context,” Social Science & Medicine (1982) 38, no. 8 (1994): 1091–1110, 10.1016/0277-9536(94)90226-7. 8042057

[16] M. E. Kruk , A. D. Gage , C. Arsenault , et al., “High‐Quality Health Systems in the Sustainable Development Goals Era: Time for a Revolution,” Lancet Global Health 6, no. 11 (2018): e1196–e1252, 10.1016/S2214-109X(18)30386-3. PMC7734391 30196093

[17] J. A. Salomon , J. A. Haagsma , A. Davis , et al., “Disability Weights for the Global Burden of Disease 2013 Study,” Lancet Global Health 3, no. 11 (2015): e712–e723, 10.1016/S2214-109X(15)00069-8. 26475018

[18] R. Team RC : A Language and Environment for Statistical Computing. Vienna: R Foundation for Statistical Computing; 2023.

[19] D. Bates , M. Mächler , B. Bolker , and S. Walker , “Fitting Linear Mixed‐Effects Models Using lme4,” Journal of Statistical Software 67, no. 1 (2015): 1–48, 10.18637/jss.v067.i01.

[20] M. Assel , D. Sjoberg , A. Elders , et al., “Guidelines for Reporting of Statistics for Clinical Research in Urology,” BJU International 123, no. 3 (2019): 401–410, 10.1111/bju.14640. PMC6397060 30537407

[21] J. P. Souza , L. T. Day , A. C. Rezende‐Gomes , et al., “A Global Analysis of the Determinants of Maternal Health and Transitions in Maternal Mortality,” Lancet Global Health 12, no. 2 (2024): e306–e316, 10.1016/S2214-109X(23)00468-0. 38070536

[22] Primary News Source . Integrating Family Planning and Maternity Services Reduces Global Maternal Mortality 2025, https://www.pharmabiz.com/NewsDetails.aspx?aid=182219&sid=2.

[23] S. Gabrysch and O. M. Campbell , “Still Too Far to Walk: Literature Review of the Determinants of Delivery Service Use,” BMC Pregnancy and Childbirth 9, no. 1 (2009): 34, 10.1186/1471-2393-9-34. PMC2744662 19671156

[24] C. Mlambo , B. Mvuyana , and B. Ntshangase , “Determinants of Maternal Mortality in Southern Africa: A Macro‐Level Analysis,” Women 3, no. 1 (2023): 132–151, 10.3390/women3010011.

