# Supplementary material for: Global Trends and Disparities in Maternal Mortality: A Longitudinal Multi‐Country Analysis
Source: Health Sci Rep. 2026 Nov 5;9(11):e73478. doi: 10.1002/hsr2.73478 (PMC13735214; doi:10.1002/hsr2.73478)
Supplement: Supplementary file 1 [file HSR2-9-e73478-s001.docx]

**Supplementary Figure S1. Distribution of residuals from the linear mixed-effects model.** The histogram shows the distribution of model residuals with an overlaid normal density curve. The residuals were approximately symmetric and centered on zero, with minor deviations in the tails.
